# Multifocal Septic Manifestations of Chronic Krokodil Abuse

**DOI:** 10.7759/cureus.107866

**Published:** 2026-04-28

**Authors:** Rashmitha Pippari, Aditya Lal Vallath, Mohsin Hamid

**Affiliations:** 1 Internal Medicine, Conemaugh Memorial Medical Center, Johnstown, USA; 2 Emergency Medicine, Conemaugh Memorial Medical Center, Johnstown, USA; 3 Critical Care, Conemaugh Memorial Medical Center, Johnstown, USA

**Keywords:** desomorphine, epidural abscess, iv drug abusers, krokodil, severe sepsis

## Abstract

This case report highlights the severe clinical course of a 30-year-old woman with a history of intravenous drug abuse, who presented to the emergency department with debilitating lower back pain. Initial evaluations revealed a critical condition, with septic emboli disseminated throughout her lungs, brain, and kidneys, likely secondary to her intravenous drug use. Further investigation suggested the involvement of krokodil (desomorphine), a potent and highly corrosive opioid, which has recently resurfaced in rural American communities due to its low cost and availability compared to fentanyl and xylazine. Krokodil, produced from codeine and toxic chemicals, causes extreme tissue damage, including ulcerations and gangrene. Radiological studies confirmed septic thrombosis, multiple septic emboli, and bilateral acute pyelonephritis, prompting immediate broad-spectrum antibiotic treatment. Despite initial stabilization, the patient’s condition escalated, necessitating a laminectomy for epidural abscess evacuation. Her treatment was complicated by withdrawal symptoms, and further bacterial cultures identified *Staphylococcus aureus* as the causative agent. The patient’s management included extended antibiotic therapy and a long-term anticoagulation regimen due to septic thrombosis. While krokodil use has been documented in other regions, including Russia and Ukraine, its prevalence in the United States remains low. Enhanced diagnostic tools and better surveillance systems are crucial to understanding and mitigating the impact of this dangerous substance.

## Introduction

This case report documents the harrowing experience of a 30-year-old woman admitted to the emergency department with debilitating lower back pain with a significant history of intravenous drug abuse. Initial assessments revealed a far more critical situation: extensive septic emboli disseminated throughout her lungs, brain, and kidneys, directly linked to her chronic intravenous drug use. However, the severity of her condition also strongly suggests the potential influence of krokodil (desomorphine), a highly corrosive and dangerous opioid that has alarmingly re-emerged in rural American communities. This substance, often cheaper and more readily available than fentanyl or xylazine combinations, is rapidly becoming a significant threat.

Krokodil, named for the scale-like, greenish skin lesions it induces, poses a particularly vicious danger. It is synthesized from codeine tablets, gasoline, paint thinner, hydrochloric acid, iodine, and red phosphorus scraped from matchboxes, resulting in a cocktail of toxic chemicals. These chemicals cause devastating damage to the body’s tissues, leading to severe skin ulcerations, gangrene, and the characteristic scale-like skin [1].

The emergence of krokodil in rural areas, where access to healthcare and addiction treatment may be limited, presents a significant public health crisis. The rapid tissue destruction, severe infections, and high mortality rates associated with krokodil highlight the urgent need for increased awareness, early intervention, and comprehensive treatment strategies. This case serves as a stark reminder of the escalating risks associated with these potent and dangerous drug combinations, emphasizing the growing prevalence of septic complications in regions historically less affected by such severe health crises.

## Case presentation

**Figure 1:**
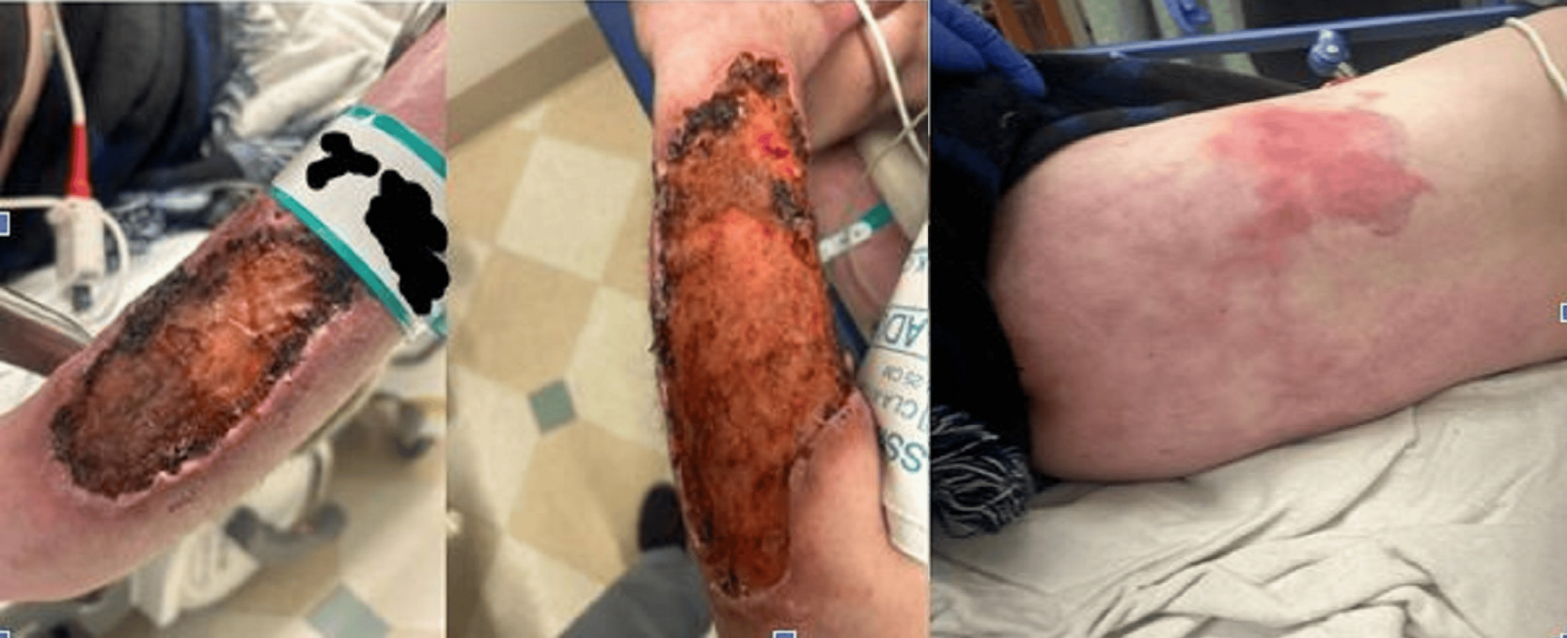
Extensive wounds on the bilateral arms and skin mottling on the back.

A 30-year-old female presented to the emergency department via ambulance with a chief complaint of back pain. She reported experiencing lower back pain for several days, which had worsened significantly on the day of presentation. She had been using a heating pad on the affected area for the preceding two to three days in an attempt to alleviate the pain. The patient reported a concern that she may have burned herself with the heating pad due to the increased severity of her pain. She denied any recent trauma or falls. She also admitted to a history of ongoing intravenous (IV) heroin and cocaine use. Additionally, the patient reported chronic skin breakdown on her arms (Figure 1), present for several months, which she attributed to the use of a drug that she initially refused to mention. On further questioning, she admitted to using IV krokodil. She denied any fever, chills, nausea, vomiting, chest pain, or shortness of breath. Her physical examination findings are summarized in Table 1.

**Table 1:** Initial physical examination findings.

Region	Description
General	Ill-appearing, in moderate distress due to back pain
Eyes	No scleral icterus
Head, eyes, ears, nose, and throat	Dry mucous membranes, poor oral hygiene
Cardiovascular	Tachycardic, regular rhythm, no murmurs. Faintly palpable peripheral pulses detectable by Doppler
Respiratory	Lungs clear to auscultation, no wheezing, rhonchi, or rales
Abdomen	Soft, non-tender, non-distended, negative Murphy sign, no rebound or guarding
Musculoskeletal	Midline tenderness noted in the lumbosacral region. Range of motion limited due to pain. Straight Leg Raise test positive bilaterally
Neurological	Awake, alert, no focal deficits
Skin	Large areas of skin breakdown on the dorsal forearms bilaterally, no active drainage, no cellulitis. Mottling of the distal extremities and lower back

**Figure 2:**
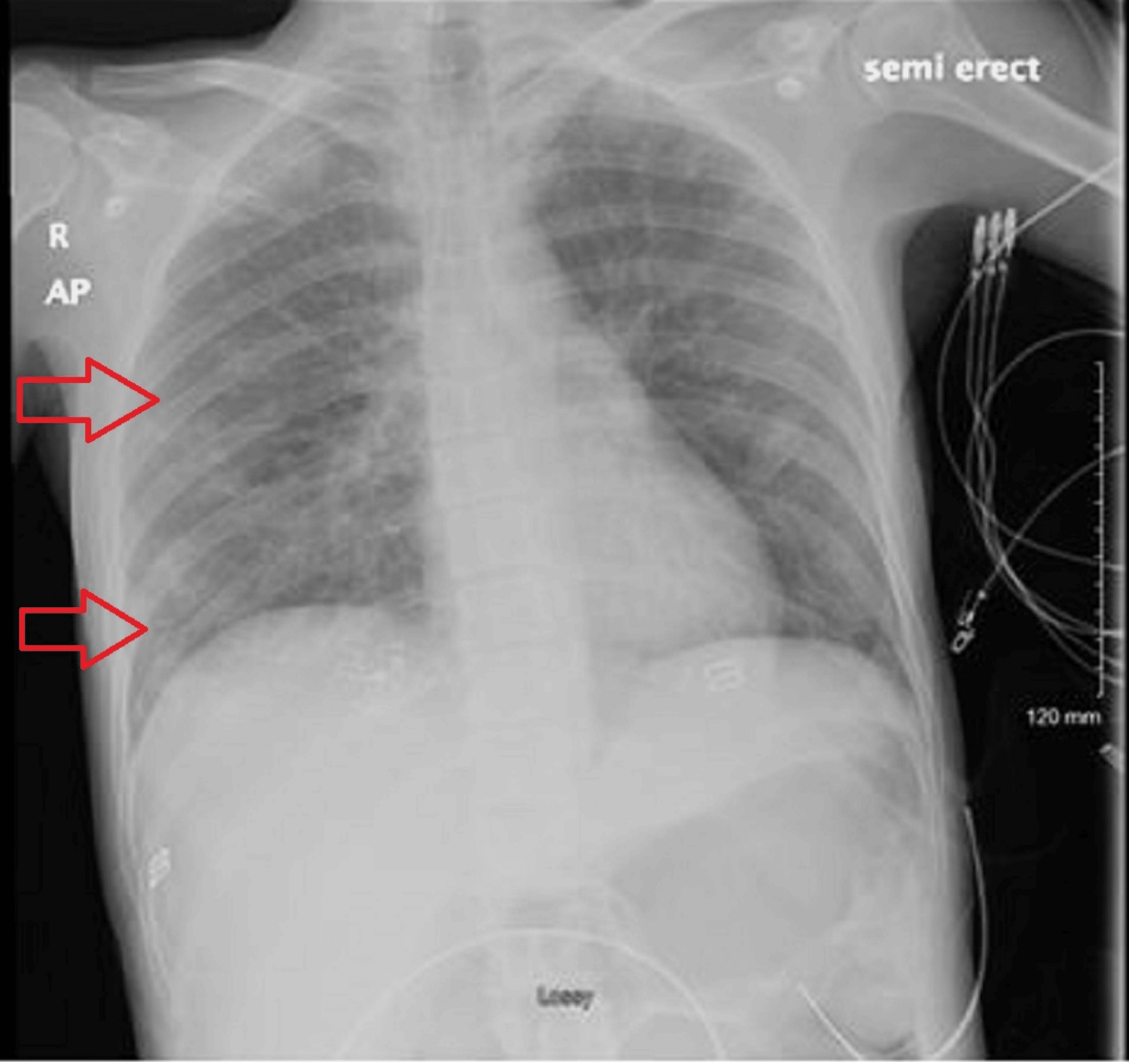
Chest X-ray showing extensive septic emboli and consolidative changes.

**Figure 3:**
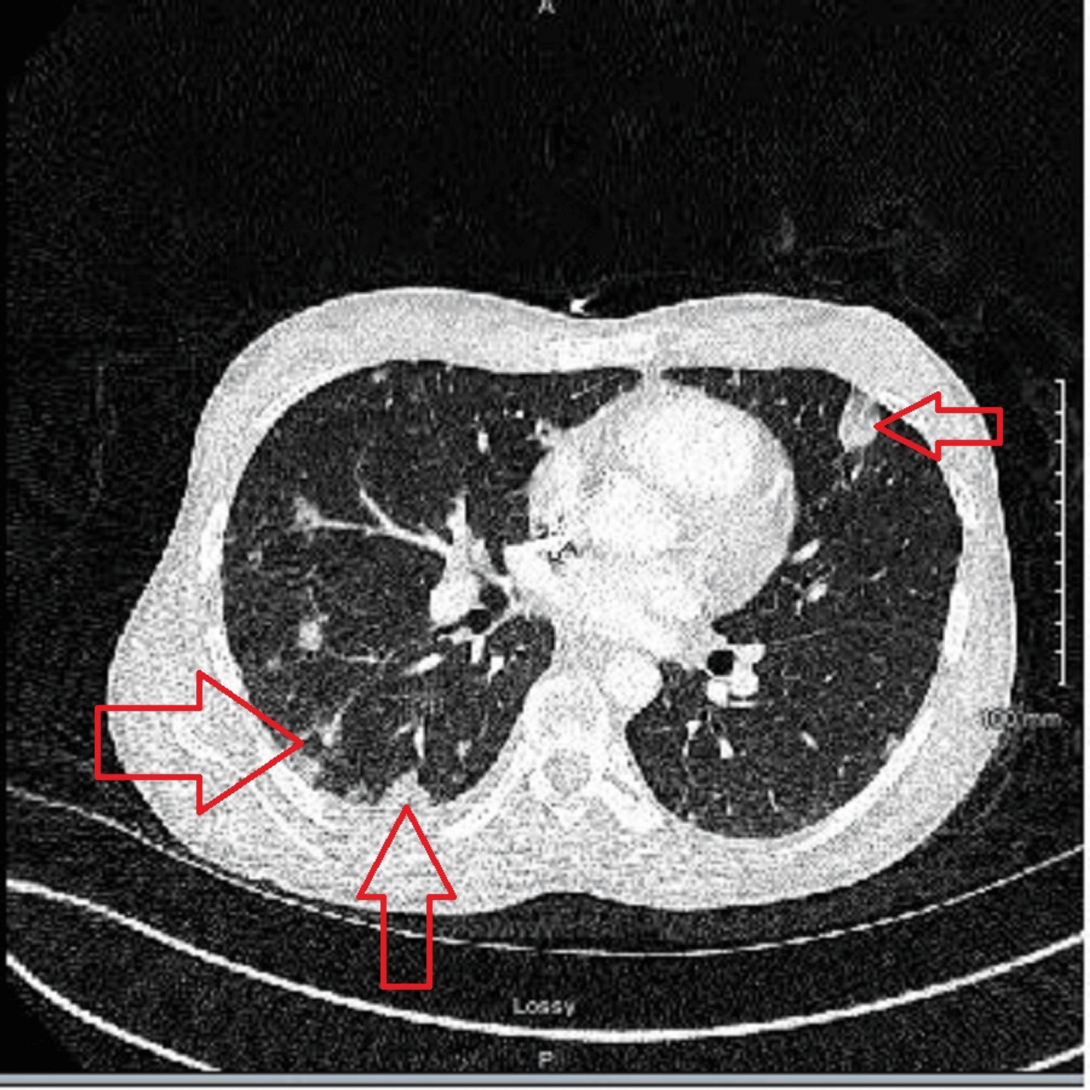
CT of the chest showing extensive septic emboli in the lungs.

**Figure 4:**
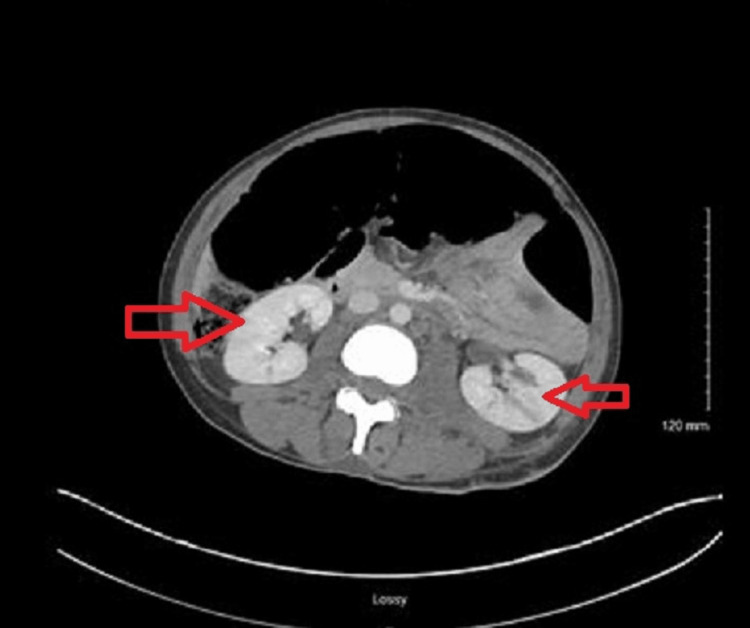
Bilateral septic emboli and extensive pyelonephritis.

Given the patient’s history of IV drug use and severe back pain, a preliminary chest X-ray (Figure 2), followed by a CT of the chest and abdomen (Figures 3, 4), was performed. Radiological studies demonstrated septic thrombosis of bilateral common iliac veins, multiple septic emboli in the lungs, and bilateral acute pyelonephritis. Complete findings are detailed in Table 2.

**Table 2:** Radiological findings.

Chest X-ray	
Pulmonary vasculature	Mild cephalization
Lung fields	Patchy airspace disease and increased interstitial markings consistent with diffuse pneumonitis. Multiple small cavitary nodules are identified, consistent with septic emboli
Pleura	No significant pleural effusion. No pneumothorax is seen
Musculoskeletal	No acute osseous abnormality is identified
CT of the chest with IV contrast (pulmonary embolism protocol)	
Lungs	The trachea and mainstem bronchi are patent and well aerated. Note is made of extensive, numerous, primarily peripheral, cavitating septic emboli throughout both lungs involving all pulmonary lobes (Figure 3). No acute bland pulmonary arterial thromboemboli are visualized. Small right pleural effusion. No pulmonary alveolar consolidation or pulmonary vascular congestion is appreciated
Cardiac	The heart is normal in size. A small pericardial effusion is demonstrated. No thoracic aortic aneurysm is noted
Skeletal	The bony thorax is intact. Specifically, no acute infectious spondylodiscitis is identified
CT of the abdomen and pelvis with IV contrast	
General	No intraperitoneal free air. Enlarged retroperitoneal lymphadenopathy is noted, which may be inflammatory or infectious
Lungs	Multiple bilateral primary peripheral scattered septic emboli at both lung bases, a few of which demonstrate mild central cavitation
Vascular	Marked septic thrombosis is present in both common iliac veins and within the inferior vena cava. The abdominal aorta is unremarkable
Kidneys	Scattered low-attenuation areas are noted in the bilateral renal cortex, consistent with bilateral acute infectious pyelonephritis (Figure 4)
Gastrointestinal tract	Extensive gas and stool distention of the colon is noted, related to a marked ileus
Pelvis	Hazy inflammation and edema are noted within the pelvic mesentery
Spine	The lumbar spine, bony pelvis, or proximal femurs are intact without evidence of acute osteomyelitis or infectious spondylodiscitis

The patient was started on broad-spectrum antibiotics, including vancomycin and piperacillin-tazobactam, and was initially given a 1 L IV fluid bolus. Initial lab results (Table 3) showed leukocytosis of 33 and lactic acidosis of 3.8 × 10³/μL, prompting additional IV fluids to complete the 30 cc/kg bolus. The patient was reassessed and updated on these results, and informed of the need for admission for IV antibiotics and further workup/management, which she agreed to. The patient was given multiple doses of Dilaudid for pain while in the emergency department. Tachycardia improved with IV fluids and pain medications. Although the patient was relatively stable from a hemodynamic standpoint, except for the tachycardia, an intensive care unit (ICU) consult was made for close monitoring due to the high risk of decompensation based on the CT findings.

**Table 3:** Laboratory values.

Category	Test	Result	Normal range	Units
Complete blood count	White blood cell count	32.36	4.0–11.0	×10³/μL
	Red blood cell count	4.25	4.5–5.9	×10⁶/μL
	Hemoglobin	11.8	13.5–17.5	g/dL
	Hematocrit	34	41–50	%
	Mean corpuscular volume	80	80–100	fL
	Mean corpuscular hemoglobin	27.8	27.0–33.0	pg
	Mean corpuscular hemoglobin concentration	35	32.0–36.0	g/dL
	Red cell distribution width	12.8	11.0–15.0	%
	Platelets	211	150–450	×10³/μL
	Mean platelet volume	11.1	7.0–11.0	fL
Comprehensive metabolic panel	Sodium	121	136–145	mEq/L
	Potassium	3.2	3.5–5.1	mEq/L
	Chloride	86	98–107	mEq/L
	Bicarbonate (CO_2_)	24	22–29	mEq/L
	Anion gap	11	7–15	mEq/L
	Calcium	8.5	8.5–10.5	mg/dL
	Glucose (fasting)	162	70–99	mg/dL
	Blood urea nitrogen	13	7–20	mg/dL
	Creatinine	0.6	0.70–1.30	mg/dL
	Estimated glomerular filtration rate	124	>60	mL/min/1.73m²
	Albumin	3.8	3.5–5.0	g/dL
	Total protein	7.2	6.4–8.3	g/dL
	Alkaline phosphatase	197	40–129	U/L
	Aspartate aminotransferase	24	10–40	U/L
	Alanine aminotransferase	28	7–56	U/L
	Total Bilirubin	0.6	0.1–1.2	mg/dL
Urine analysis	Color	Yellow	Yellow/Straw	N/A
	Clarity	Cloudy	Clear	N/A
	pH	6.5	4.5–8.0	N/A
	Specific gravity	1.045	1.005–1.030	N/A
	Glucose (urine)	250	Negative	mg/dL
	Bilirubin (urine)	Negative	Negative	mg/dL
	Ketones	Negative	Negative	mg/dL
	Urobilinogen	1	0.2–1.0	mg/dL
	Leukocytes	Small	Negative	N/A
	Nitrite	Positive	Negative	N/A
	Blood	Small	Negative	N/A
	Protein	100	Negative	mg/dL
	Red blood cell count (microscopic)	0–2	0–2	/hpf
	White blood cell count (microscopic)	11–20	0–5	/hpf
	Bacteria	2+	Negative	/hpf
	Squamous epithelial	11–20	0–15	/hpf
	Hyaline casts	0-8	0–5	/lpf
Other	Troponin I (high sensitivity)	<3	<0.04	ng/mL
	Lactic acid (lactate)	3.8	0.5–2.2	mmol/L
	HCG (serum)	Negative	Negative	mIU/mL

In the ICU, the patient was closely monitored. Preliminary report on blood cultures showed Gram-positive cocci in clusters, with the microbial gene panel indicating likely methicillin-sensitive *Staphylococcus aureus*. She was empirically treated for bacterial endocarditis as she met Duke’s criteria. Her initial transthoracic echocardiogram showed no vegetations. Given the concern for infective endocarditis, this was later confirmed by a transesophageal echocardiogram. Due to extensive septic emboli in multiple organs, vancomycin and piperacillin-tazobactam were continued. During her initial ICU stay, however, she exhibited signs and symptoms of withdrawal, leading to combativeness and the removal of her IV lines. Her agitation was managed with a dexmedetomidine and midazolam infusion, achieving a Richmond Agitation-Sedation Scale score of -1.

**Figure 5:**
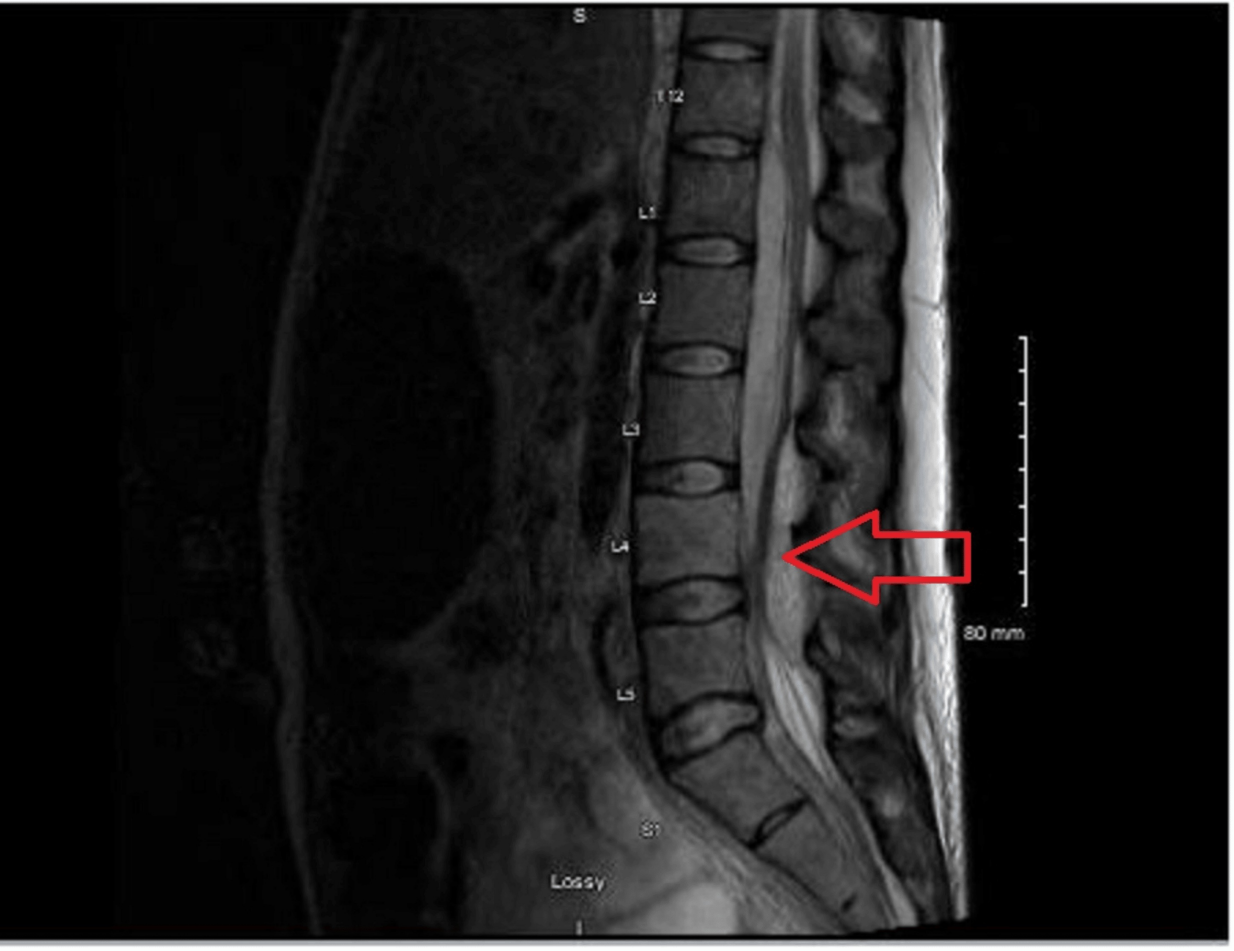
T2 sagittal view MRI of the lumbar spine. A large epidural abscess extended from T12 to L4, with the largest measurement of 11 × 22 mm at L1-2, causing severe central canal stenosis and posterior displacement of the thecal sac. Another large abscess was present from L4 to L5, measuring 11 × 10 mm, resulting in severe central canal stenosis and anterior displacement of the thecal sac.

**Figure 6:**
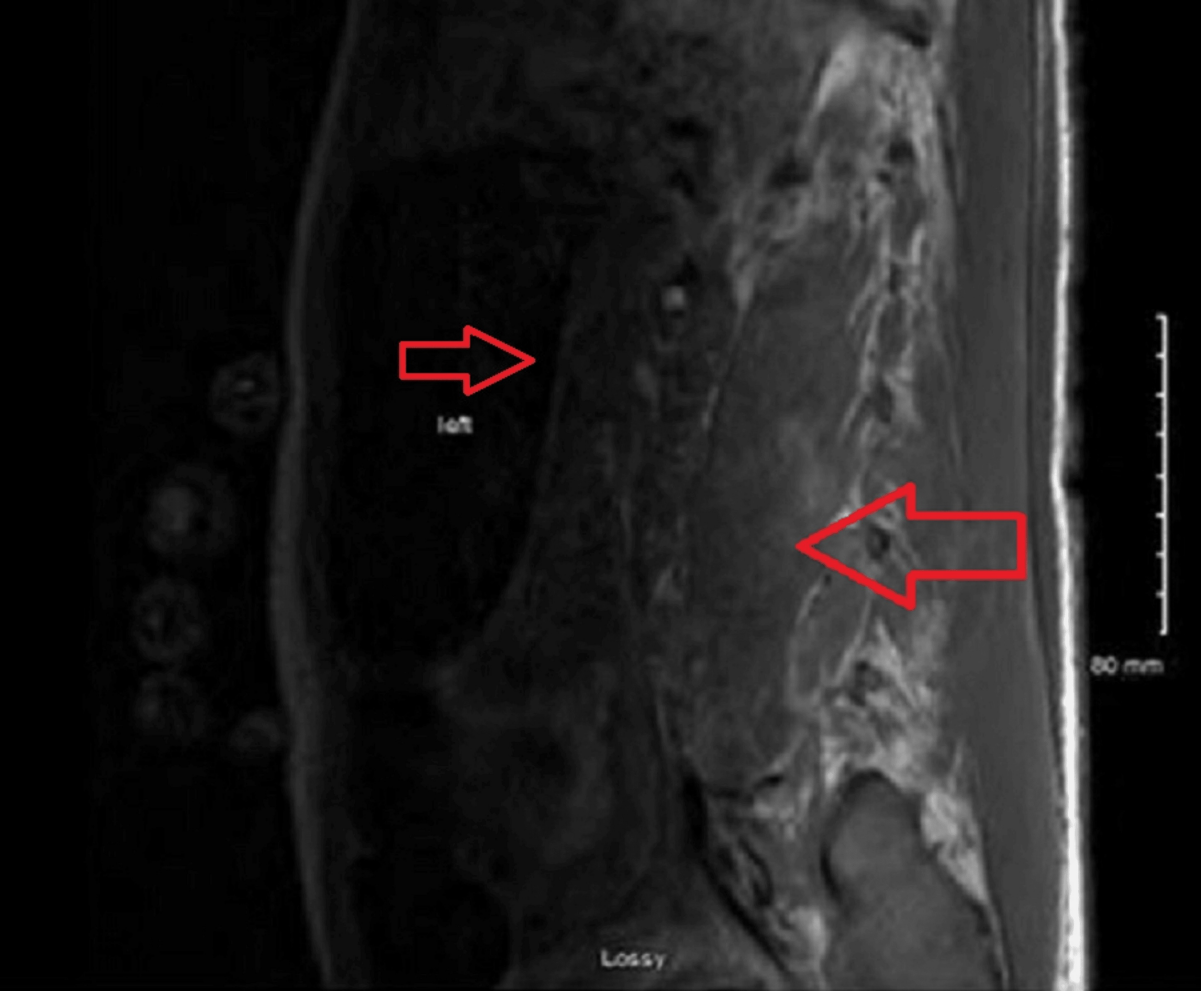
T1 sagittal view of the lumbar spine showing myositis of the paraspinal muscle with a left-sided paraspinal fluid collection.

On the third day of hospitalization, the urine culture showed significant bacterial growth, >100,000 CFU/mL of *Staphylococcus aureus* and 10,000-25,000 CFU/mL of mixed organisms. Blood cultures from both aerobic and anaerobic bottles also revealed the presence of *Staphylococcus aureus*, with Gram-positive cocci in clusters observed on the Gram stain. Given concerns about inadequate antibiotic coverage, the infectious disease team switched the initial antibiotics to cefazolin and ertapenem. An MRI was also ordered to assess for a potential epidural abscess. MRI of the brain and cervical spine did not reveal any infectious foci; however, MRI of the thoracic spine and lumbar spine revealed extensive infective pathology (Table 4; Figures 5, 6).

**Table 4:** MRI findings.

MRI with and without contrast	
Head	
Impression	No abnormal brain or abnormal meningeal enhancement
Cervical spine	
Impression	No evidence for focal herniated nucleus pulposus, significant foraminal or spinal stenosis. Normal enhancement of the cervical cord is visualized. Mild soft-tissue edema in the paraspinal musculature in the upper thoracic spine
Thoracic spine	
Impression	Subtle bone edema identified in the T12 and L1 vertebral bodies may represent early osteomyelitis. Epidural abscess seen on the margin of the study extending from T12 inferiorly. No evidence for discitis
Lumbar spine	
Meninges	Diffuse enhancement within the leptomeninges as well as within the epidural space is consistent with inflammatory and infectious change
Epidural space	A large epidural abscess seen extending anteriorly from T12 through L4 is identified, largest at the L1-2 level, measuring 11 × 22 mm in transverse dimension. Severe central canal stenosis is seen at this level with posterior displacement of the thecal sac. A large epidural abscess posteriorly from L4 to L5, which measures 11 × 10 mm in diameter, and causes severe central canal stenosis at L4-5 and anterior displacement of the thecal sac
Vertebrae	Osteomyelitis from T12 through S1 is suspected, with edema in the osseous structures identified. No evidence for compression deformity
Paraspinal musculature	Significant inflammatory change and infectious change with myositis with a left-sided paraspinal fluid collection measuring 22 × 16 mm in size external to the left L4-5 foramen. Several other smaller paraspinal fluid collections in the paraspinal musculature were identified

Neurosurgery was consulted and, on day five of hospitalization, performed an L3-4 laminectomy for epidural abscess evacuation. On the sixth day of hospitalization, the patient’s drain culture revealed moderate growth of *Staphylococcus aureus*, a significant finding indicating ongoing infection. The infectious diseases team recommended continuing the existing antibiotic regimen, emphasizing the need for a prolonged course of at least eight weeks, calculated from the date of the first negative blood cultures, to ensure eradication of the deep-seated infection.

On day eight, antibiotic therapy was adjusted, discontinuing ertapenem and maintaining cefazolin at a dose of 2 g every eight hours while the patient remained inpatient. The plan was for a prolonged course of IV antibiotics, lasting a minimum of eight weeks and potentially extending to ten weeks, to address the complex infection. Furthermore, given the presence of septic thrombosis in the inferior vena cava and iliac veins, long-term anticoagulation with a direct oral anticoagulant for up to six months was deemed necessary to prevent further thrombotic complications.

The patient was subsequently transferred to the medical floor, where she continued to receive IV antibiotics as per the infectious diseases team’s recommendations. She also received multidisciplinary care, involving neurosurgery, psychiatry, and plastic surgery, to address her various comorbid conditions.

On day 16, a peripherally inserted central catheter (PICC) line was placed to facilitate long-term antibiotic administration. This decision was made in response to the patient’s family’s assurance that they could supervise her long-term antibiotic infusions, mitigating the risk of IV drug use. Concurrently, the inpatient psychiatric team made significant progress in managing the patient’s withdrawal symptoms and pain. A long-term opioid dependence treatment facility was identified and accepted the patient.

However, on day 35, the patient absconded from the hospital with the PICC line still in place. The local police were notified and apprehended the patient the following day. They reported finding her intoxicated, and she admitted to resuming IV drug use. Upon her return to the emergency department, the PICC line was removed. The patient declined further treatment and left against medical advice.

## Discussion

Desomorphine, known on the streets as krokodil, is a potent opioid derivative of codeine that exerts sedative and analgesic effects and carries a high risk of addiction [1]. The street name “krokodil” originates from the Russian word for crocodile [2], a reference to the distinctive scale-like skin that can develop in individuals who use the drug over extended periods [1]. The drug is also referred to as “Russian Magic,” alluding to the brief duration of the euphoric intoxication it produces, and “Poor Man’s Heroin,” a moniker that reflects its lower cost compared to heroin [1]. Chemically, desomorphine is a semi-synthetic analogue of morphine, first synthesized in the 1930s [1].

Desomorphine’s mechanism of action is similar to that of other opioids, with an estimated 10 to 15 times the strength of morphine contributing to its significant addictive potential. The drug acts rapidly, within two to three minutes of administration, but typically lasts less than two hours. The short-lived euphoric effects compel users to engage in frequent re-dosing to sustain the high and avoid the onset of withdrawal symptoms [1]. This pattern of rapid onset and short duration fosters rapid physical dependence and a high likelihood of addiction [1].

Krokodil is commonly manufactured illicitly in domestic settings using codeine, which can be sourced from both prescription and over-the-counter medications [3]. The synthesis involves the use of readily available but highly toxic additives such as gasoline, paint thinner, lighter fluid, iodine, hydrochloric acid, and red phosphorus obtained from matches. These dangerous chemicals are frequently not entirely removed during the “cooking” process [4]. The precise chemical composition of krokodil can vary considerably depending on the products used in its makeshift manufacture [5,6]. This includes various opioid alkaloids along with elevated concentrations of the processing chemicals, which are responsible for the localized and systemic injuries observed in users [7]. Furthermore, the resulting mixture is often highly acidic with a pH below 3, which significantly increases the risk of tissue damage at the injection site [3]. Toxic byproducts such as iron, zinc, and lead can also be present in the final concoction [6]. The lack of quality control in its synthesis means that users are exposed to unpredictable levels of desomorphine and varying concentrations of harmful contaminants, substantially increasing their risk of overdose and severe toxic reactions [4-6]. The cycle of rapid addiction coupled with the contamination from byproducts of its crude manufacturing process likely exacerbates the severe health consequences observed in krokodil users [7,8].

The initial reports of krokodil use emerged in Siberia, Russia, in 2002 [6]. From its origins, the drug primarily spread through various European countries before its presence was noted in the United States [9]. The countries most significantly affected by krokodil use appear to be Russia and Ukraine [2]. However, instances of its use and related injuries have also been reported in Georgia, Germany, Kazakhstan, the Czech Republic, France, Belgium, Sweden, and Norway [2]. In Russia, the scale of krokodil use was substantial, with estimates suggesting as many as one million users by 2010 or 2011 [4,5]. In 2013, the prevalence of krokodil use in Russia and Ukraine was estimated to be between 5% and 7% among individuals who inject drugs [6].

The emergence of krokodil as a drug of abuse is closely linked to its status as a cheaper alternative to heroin [1]. The primary reason for its lower cost is the relative ease of its homemade production using readily available codeine and other inexpensive ingredients [4]. Specifically, in Russia, the cost of producing desomorphine from over-the-counter codeine was significantly lower than the cost of purchasing heroin on the street [4]. Additionally, periods of heroin shortage in Russia around 2010 are believed to have further contributed to the increased use of krokodil as a substitute, illustrating how drug regulation can significantly impact substance abuse patterns [9,10]. The subsequent decrease in reported cases following the implementation of codeine restrictions suggests that controlling the availability of precursor substances can be an effective strategy for mitigating harm.

Starting around September 2013, numerous unconfirmed news reports began to surface regarding the alleged use of krokodil in the United States and Canada, capturing significant attention from the media [10]. These reports often employed sensational language, dubbing it the “drug that eats junkies,” “Russia’s deadly designer drug,” and the “flesh-eating” or “flesh-rotting” drug, frequently accompanied by disturbing images depicting the severe physical effects on users [2]. Specific reports originated from Arizona, Illinois, and Oklahoma, further fueling public concern [10]. However, a contrasting perspective emerged, with some considering the prevalence of krokodil in the United States to be more of an urban legend than a well-substantiated medical reality, and findings from the Drug Enforcement Administration (DEA) presented a different picture [9].

In contrast to the media reports and official statements, the only instance of desomorphine identification by the DEA’s National Forensic Laboratory Information System (NFLIS) was in 2004, when two samples were found to contain the substance [6]. Furthermore, the DEA’s 2014 Drug Threat Assessment Report explicitly stated that “there are no confirmed cases of krokodil abuse in the United States.” Reports of its presence in Canada have been disproven by the Canadian Centre of Substance Abuse [11]. As of July 2019, the DEA stated that they had not identified any samples of krokodil (desomorphine) in their analyses since 2004 [1]. It is important to note that desomorphine has been classified as a Schedule I substance in the United States since 1936, a designation that indicates a high potential for abuse and no currently accepted medical use [3]. Despite the lack of recent identifications, the DEA maintains vigilance regarding emerging drug trends and potential threats to public health [6].

Data from the Centers for Disease Control and Prevention (CDC) also do not prominently feature krokodil in their reports concerning drug overdose deaths [9]. The CDC’s data primarily focuses on the broader opioid crisis, with a significant emphasis on fentanyl and its analogues [11,12]. Recent CDC reports have highlighted the increasing threat posed by fentanyl mixed with xylazine, another substance that has been associated with severe skin wounds in users [13]. CDC data from 2023 indicate a decline in overdose deaths involving illegally manufactured fentanyl in the Northeast, Midwest, and South regions, but an increase in the West [14]. The CDC also tracks emergency department visits related to opioid misuse, providing a general overview of the opioid crisis [15].

In many instances, reports of suspected krokodil use in the United States have not been substantiated by toxicology or laboratory analyses. While desomorphine can be detected in biological samples such as blood within a few hours and urine within two to three days after use, routine testing for this specific substance is not typically available in clinical settings. Furthermore, the drug’s relatively short half-life can complicate its detection [3]. Without widespread testing capabilities, it is difficult to confirm suspected cases and track trends effectively, potentially leading to an incomplete understanding of the issue. The similar physical manifestations associated with xylazine use might lead to assumptions of krokodil use, particularly given the notoriety of krokodil’s effects [13].

Medical case reports and studies, while limited in the United States, offer valuable insights into the clinical presentation of krokodil abuse. One case report detailed a 23-year-old woman with a history of IV drug abuse who developed extensive ulcerations [16]. In New York, a case was reported of a 27-year-old female who presented with bilateral arm ulcerations resulting from injecting krokodil over a period of five to six months [17]. A 41-year-old man admitted to a hospital in Ottawa with necrotic leg ulcers was later found to have been injecting krokodil [18]. Another case in Spain reported a novel case of oral ingestion of krokodil resulting in gastrointestinal symptoms such as nausea and vomiting [19]. In another case, a 19-year-old male with a history of krokodil abuse presented with a non-healing skin ulcer, cardiac arrhythmia, and deep vein thrombosis, suggesting potential cardiovascular complications associated with the drug [7]. One commentary discussed a case where skin lesions were initially implicated as being caused by krokodil but argued that, without analytical confirmation, other causes, such as heroin adulterated with levamisole, could not be ruled out [11].

While the number of medical case reports from the United States remains limited, they provide crucial evidence of the devastating health consequences associated with krokodil use, even in isolated instances. These cases illustrate the clinical presentation of krokodil abuse and emphasize the importance for healthcare professionals to consider this potential diagnosis in patients presenting with severe, unexplained skin and soft-tissue infections, particularly in those with a history of drug use.

The apparent contradiction between the significant media attention surrounding krokodil and the lack of widespread confirmed cases according to official sources like the DEA suggests a potential disconnect between public perception, often shaped by sensationalized media narratives, and the actual prevalence of the drug in the United States. The absence of confirmed cases in DEA laboratory analyses since 2004 indicates that either the drug is not commonly used or that suspected cases may be attributable to other factors or substances. The overwhelming focus of official health agencies like the CDC on the broader opioid crisis, driven significantly by fentanyl, likely leads to less attention being given to the specific issue of krokodil in national surveillance data. This is because krokodil appears to have a much smaller impact when compared to the widespread problem of opioid abuse in the United States. While krokodil usage may have increased to some extent, it has not reached the same levels of prevalence as heroin. Heroin use in the United States is estimated to affect nearly one million individuals annually [20,21]. The United States continues to grapple with a significant opioid epidemic, primarily driven by the misuse of prescription opioids and the widespread availability of illicitly manufactured fentanyl [22,23].

The spread of krokodil to other European countries indicates the potential for drug trends to transcend national borders, particularly among vulnerable populations seeking more affordable alternatives to traditional opioids. Migration and the interconnectedness of illicit drug markets can facilitate the introduction of new substances into different regions, even if the overall prevalence remains low. Furthermore, the significant role of economic factors in the adoption of krokodil highlights that individuals in resource-limited settings who are grappling with opioid addiction may be more inclined to use cheaper, albeit considerably more dangerous, alternatives. Globally, krokodil often emerges as a drug of last resort in conditions of extreme poverty, economic crisis, or conflict, making zones like war-affected Ukraine potentially vulnerable to its use. The full-scale Russia-Ukraine war since 2022 has distinctly altered the drug landscape in the region. According to the Global Initiative Against Transnational Organized Crime, the war has fueled a dramatic increase in Ukraine’s overall synthetic drug market, reportedly the largest increase globally between 2021 and 2023. This surge was driven by factors including combat stress, displacement, and organized crime, adapting to wartime conditions. Krokodil use among both soldiers and civilians has risen year after year [24-26].

Several factors may have influenced the observed trends in krokodil use in the United States. The regulatory environment surrounding codeine in the United States may have limited the widespread production of krokodil [1]. The established market for heroin and the increasing dominance of fentanyl, which is often cheaper and more potent, might have reduced the demand for krokodil as an alternative, particularly given the severe health risks associated with its use [4]. Public awareness campaigns highlighting the devastating health consequences of krokodil might also have acted as a deterrent for potential users [9]. If krokodil use is indeed rare, it may not register significantly in national-level data on drug-related hospital encounters. Alternatively, the lack of widespread testing for desomorphine could mean that cases are being categorized under broader diagnoses, such as skin infections or general opioid abuse, without specific mention of krokodil. Given the severe health consequences associated with krokodil, it is plausible that when cases do occur, they are likely to result in emergency room visits and hospital admissions due to serious complications such as severe infections, extensive necrosis, and potential damage to internal organs. The “flesh-eating” nature of the drug suggests that users would eventually seek medical attention for the severe tissue damage, although this may be delayed due to fear or other factors. Enhancing diagnostic capabilities to specifically identify desomorphine and improving data collection methods to track krokodil-related hospital encounters would be beneficial in gaining a more accurate understanding of its prevalence and impact on the healthcare system, even if current numbers appear to be low. Non-profit and harm reduction groups play a vital role in providing education about the risks of krokodil and advocating for access to treatment and support services for individuals struggling with substance use disorders, regardless of the specific drug involved. These organizations often work directly with vulnerable populations and aim to reduce harm and promote recovery through practical information and resources [27,28]. Improved surveillance could help monitor for any potential future increase in krokodil use and inform public health responses if necessary.

## Conclusions

Desomorphine, notoriously known as krokodil, is a potent, illicitly synthesized opioid derived from codeine and other toxic chemicals, infamous for causing severe tissue damage. The primary opioid crisis in the United States continues to be driven by fentanyl and heroin, with factors such as stricter codeine regulation possibly limiting krokodil’s spread. Although the threat level in the United States seems low, the devastating health consequences necessitate ongoing vigilance, better surveillance, and research.
